# Supplementary material for: CRISPR/Cas9 Mutagenesis through Introducing a Nanoparticle Complex Made of a Cationic Polymer and Nucleic Acids into Maize Protoplasts
Source: Int J Mol Sci. 2023 Nov 9;24(22):16137. doi: 10.3390/ijms242216137 (PMC10671792; doi:10.3390/ijms242216137)
Supplement: Supplementary file 1 [file ijms-24-16137-s001.zip › ijms-2672507-supplementary.pdf]

SUPPLEMETARY DATA

Supplementary Figure S1. PCR amplification of the green enhanced fluorescence transgene in order to verify the CRISPR mediated gene editing via Sanger sequencing.

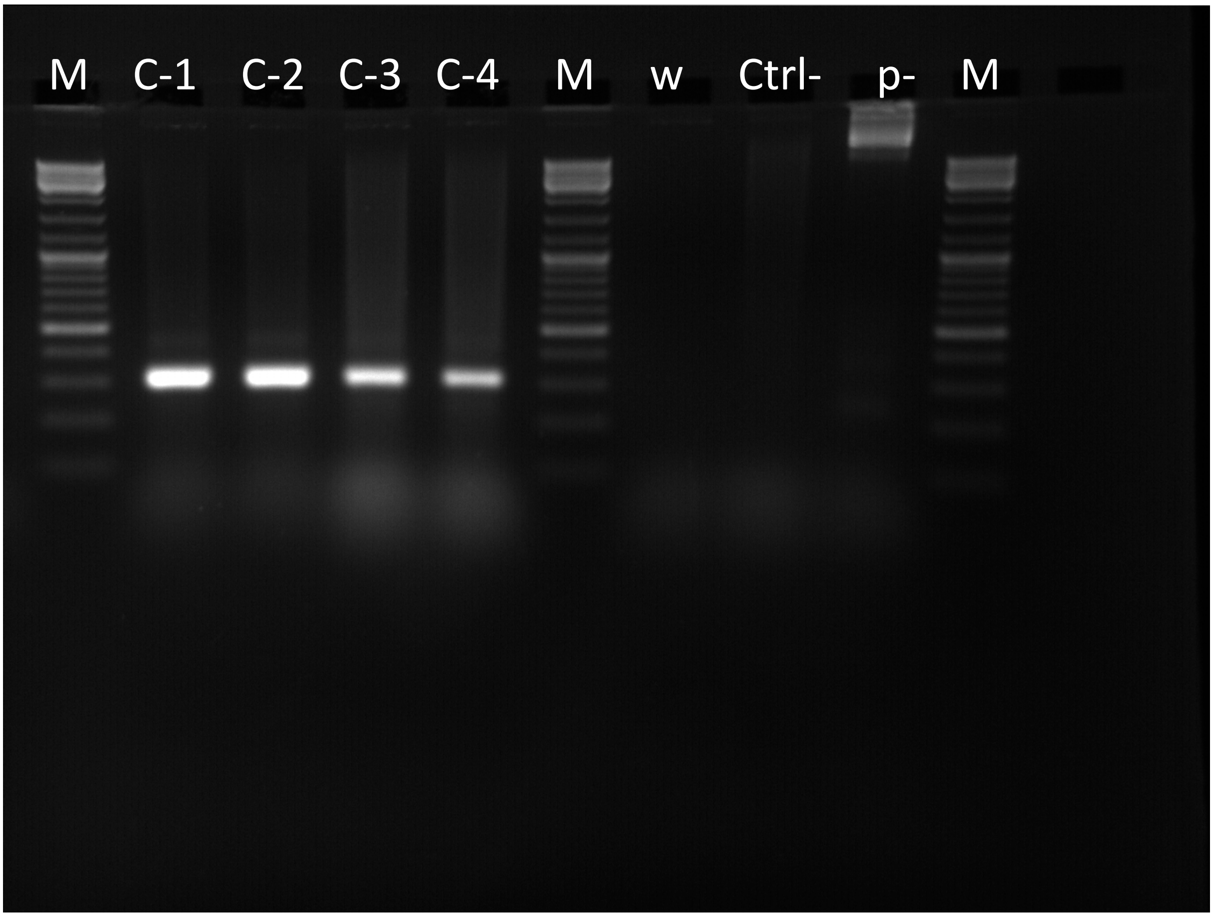

M: DNA ladder (Thermo Scientific™ GeneRuler DNA Ladder Mix, SM#0331), C-1, C-2, C-3, C-4: Clone–1-4., w: water blank. Ctrl–: negative control maize DNA, p-: plasmid negative control DNA. 1 % agarose gel electrophoresis.

PCR amplifications were performed with the following EGFP gene-specific primers: GFP_Forward: 5′-ccaaccacgtcttcaaagca-3′, GFP_Reverse: 5′- cggtggtgcagatgaacttc -3′. Phusion Hot Start II High Fidelity DNA Polymerase (Thermo Scientific) was used for the PCR reactions with the following cycle conditions: 1. Initial denaturation: 98 °C for 3 min. 2. Denaturation: 98 °C for 10 s. 3. Annealing: 67 °C for 30 s. 4. Extension: 72 °C for 20 s. (2–4 steps: 30 cycles) 5. Final extension: 72 °C for 10 min. Fragments of 317 nucleotides were used for Sanger sequencing.
